# DCTclock: Clinically-Interpretable and Automated Artificial Intelligence Analysis of Drawing Behavior for Capturing Cognition

**DOI:** 10.3389/fdgth.2021.750661

**Published:** 2021-10-15

**Authors:** William Souillard-Mandar, Dana Penney, Braydon Schaible, Alvaro Pascual-Leone, Rhoda Au, Randall Davis

**Affiliations:** ^1^Digital Cognition Technologies, Boston, MA, United States; ^2^Linus Health, Boston, MA, United States; ^3^Computer Science and Artificial Intelligence Laboratory, Massachusetts Institute of Technology, Cambridge, MA, United States; ^4^Department of Neurology, Lahey Hospital and Medical Center, Boston, MA, United States; ^5^Deanna and Sidney Wolk Center for Memory Health, Arthur and Hinda Marcus Institute for Aging Research and Hebrew SeniorLife, Boston, MA, United States; ^6^Department of Neurology, Harvard Medical School, Boston, MA, United States; ^7^Guttmann Brain Health Institute, Institut Guttmann de Neurorehabilitació, Universitat Autonoma de Barcelona, Barcelona, Spain; ^8^Departments of Anatomy & Neurobiology, and Neurology and Framingham Heart Study, Boston University School of Medicine, and Department of Epidemiology, Boston University School of Public Health, Boston, MA, United States

**Keywords:** artificial intelligence, cognition, Alzheimer's disease, dementia, DCTclock, clock drawing test, behavior analysis

## Abstract

Developing tools for efficiently measuring cognitive change specifically and brain health generally—whether for clinical use or as endpoints in clinical trials—is a major challenge, particularly for conditions such as Alzheimer's disease. Technology such as connected devices and advances in artificial intelligence offer the possibility of creating and deploying clinical-grade tools with high sensitivity, rapidly, cheaply, and non-intrusively. Starting from a widely-used paper and pencil cognitive status test—The Clock Drawing Test—we combined a digital input device to capture time-stamped drawing coordinates with a machine learning analysis of drawing behavior to create DCTclock™, an automated analysis of nuances in cognitive performance beyond successful task completion. Development and validation was conducted on a dataset of 1,833 presumed cognitively unimpaired and clinically diagnosed cognitively impaired individuals with varied neurological conditions. We benchmarked DCTclock against existing clock scoring systems and the Mini-Mental Status Examination, a widely-used but lengthier cognitive test, and showed that DCTclock offered a significant improvement in the detection of early cognitive impairment and the ability to characterize individuals along the Alzheimer's disease trajectory. This offers an example of a robust framework for creating digital biomarkers that can be used clinically and in research for assessing neurological function.

## Introduction

Early detection and diagnosis of cognitive decline is critical to the development and deployment of novel therapeutic interventions for patients with dementia due to Alzheimer's disease (AD) and other neurodegenerative diseases. Clinical detection currently largely depends on reported complaints from patients or their family, or impaired performance on standard cognitive screening tasks such as the Mini Mental State Examination (MMSE) (1) or the Montreal Cognitive Assessment (MoCA) (2). These tests have been shown to be, relatively insensitive to milder forms of impairment and require administration and hand-scoring by trained administrators, which can lead to subjectivity in both scoring and interpretation. Computerized tests, such as CANTAB Mobile (3) and Cogstate Brief Battery (4) have made significant strides in tackling these problems by automating administration and scoring processes. These tests, however, measure behaviors that are more unnatural and distant from daily living, which, combined with lengthier testing sessions, can limit the broad use of the technology, especially in older individuals. Cerebrospinal fluid and positron emission tomography (PET) imaging– gold standard diagnostic indices of earliest stage disease in neurological disorders such as AD—remain costly and invasive, have suboptimal accuracy in predicting cognitively related impairment, and provide limited clinical information for addressing patient and family concerns.

Pathology associated with AD and other neurodegenerative diseases is present for years, often decades, prior to diagnosis, and diagnosis at the point of overt clinical manifestations may be too late to meaningfully impact outcome and prognosis (5). Thus, there is not only a need for early diagnosis but also for screening and detection of abnormalities in otherwise asymptomatic individuals. To that end, digital markers might be ideally suited as rapid, user-friendly, and objective screening tools for detecting and tracking subtle cognitive change, and as differential diagnosis tools that provide clinically useful and patient-centered information to inform treatment. These characteristics would enable the idea of a new “vital sign” for the brain.

As a simple paper and pencil test used for over 50 years, the Clock Drawing Test (CDT) is quick and easy to administer, non-invasive and inexpensive, yet provides valuable clinical information and diagnostic utility (6). The test instructions first ask the subject to draw, on a blank sheet of paper, a clock showing 10 min after 11 (Command clock), and then asks them to copy a pre-drawn clock showing that time (Copy clock). Successful completion of the test requires a variety of cognitive functions, including memory (working memory, visual memory, semantic memory), executive function, selective and sustained attention, visuospatial abilities, auditory comprehension, and motor control (7). It has been a useful screening tool to differentiate normal elderly individuals from those with cognitive impairment, and has been effective in helping to diagnose dementia, Parkinson's disease, and other conditions (8).

However, the utility of the traditional CDT is reduced by a lack of consistency between diverse scoring systems, the complexity and often imprecise scoring criteria, and inter-rater unreliability due to the subjective nature of some scoring criteria (9). Additionally, clinical assessment generally considers only the final drawing, often with no evaluation of the drawing process. While more impaired patients may make significant errors in their drawings, subtly impaired patients may produce a final drawing that appears normal or near-normal and are thus at risk for having early cognitive change missed by the clinician. In contrast, analyzing the entire drawing process offers better opportunity to capture early change by revealing subtle behaviors that precede impairment such as decision-making latencies, compensatory strategies, and psycho-motor issues not visible in the final drawing.

To address these issues, a first digital version of the CDT was created by using a digitizing pen to capture the entire drawing process—both spatial and temporal data—while keeping the well-known administration procedures for the clock drawing test administration standard. The digitizing pen works as a standard ballpoint, enabling drawing on a paper and avoiding the use of a digitizing tablet, which is less natural for the elderly. From a digitally captured CDT performance, two human raters independently classified the digital pen coordinates into drawing components (i.e., digits, hands), with a third reviewer for resolving any differences. This enabled the creation and computation of novel drawing measures from these classifications, such as between pen stroke latencies and drawing velocities. Using these novel measurements, classification models were developed for the detection of cognitive impairment, with a focus on neurodegenerative disorders such as AD and Parkinson's disease, showing significant improvements over existing manual scoring systems for the CDT (10). These classification models were then further refined using clinician input to make the classification results more transparent and interpretable to clinicians, with only a small trade-off in accuracy (10). Prior research has shown that patients have longer total drawing times for completing the CDT task after total knee arthroplasty with general anesthesia (11) and during normal aging (12), and that specific process latencies during the drawing can reveal deficits in processing speed and decision making in subjects with Multiple Sclerosis (13). Previous work has also shown that the order of clock component placement in non-demented depressed older adults is associated with cognitive and brain structural connectome differences (14).

Despite promising results, this first version of the digital CDT suffered from significant limitations. First, it required lengthy human review of the drawings to classify the pen strokes into symbols, necessary to extract clinically meaningful features, rendering the test time-consuming, prone to human error, and unable to provide immediate, live results to a clinician or patient. Second, more validation data was required to demonstrate overall test accuracy, benchmark performance against other commonly-used tests, and understand clinical benefits. Third, the software and hardware of the system was not constructed to scale beyond research use, and there was a clear need to convert it into an easily-deployable platform. These issues limited the digital CDT to research use and made it impossible to deploy clinically or at scale.

The present study tackles the above limitations, summarizing the development and validation of DCTclock, a fully automated analysis of the drawing process and final drawing from the digital CDT, as a screening tool for cognitive impairment. We describe how the algorithms were developed, the resulting clinical scores, and the validation findings based on comparisons between AD and aMCI clinical samples and a community-based normal aging population. A separate validation study of this technology in pre-clinical AD individuals has already shown that DCTclock is strongly correlated with tau and amyloid loads on PET (15). DCTclock is FDA-cleared to market as a computerized cognitive assessment aid, offering a sensitive and scalable early detection method for cognitive health and dementia.

## Materials and Methods

### Compliance With Ethical Regulations

All procedures followed the ethical guidelines of the 1975 Declaration of Helsinki and data was collected under IRB approved protocols at both sites–Framingham Heart Study and Lahey Hospital and Medical Center. Data analysis herein was conducted under an IRB approved protocol at Digital Cognition Technologies/Linus Health (IRB Tracking Number 20160721).

### Study Participants

Since 2005, 1,560 subjects from an outpatient Neurology service at Lahey Hospital and Medical Center (LH) have been administered DCTclock. Subjects were administered a subset of the following neuropsychological tests within 2 weeks of DCTclock: MMSE, MoCA, Repeatable Battery for the Assessment of Neuropsychological Status (RBANS) (16), Halstad-Reitan Trail Making Test (Trails) A and B (17), Hooper Visual Organization Test (HVOT) (18), Boston Naming Test (BNT) (19), and Beck Depression Inventory (BDI) (20). All individuals had documented medical and cognitive status including medical, neurological, surgical, and psychiatric conditions based on consensus diagnosis. Consensus diagnosis was derived from imaging, neuropsychological testing, and medical record review, independently of DCTclock and MMSE performance. Three groups of subjects aged 55+ were selected from this population based on consensus diagnosis: a general cognitively impaired group (*N* = 791), and subsets of individuals diagnosed with amnestic mild cognitive impairment (aMCI; *N* = 113) and dementia due to probable AD (*N* = 128).

Beginning in 2011, DCTclock was administered as part of the standard cognitive assessment battery to participants enrolled in the Framingham Heart Study (FHS) cognitive aging and dementia studies, an established longitudinal epidemiological research program. FHS has conducted on-going surveillance for incident dementia since 1976 (21) and incident stroke since the study inception in 1948 (22). DCTclock was administered to participants in the Original, Offspring, New Offspring Spouses, and OmniGeneration 1 cohorts (*N* = 2,309). For this study, participants were excluded if aged below 55 (*N* = 610), and diagnosed with prevalent dementia and/or stroke (*N* = 76). Additionally, examiner impression of possible cognitive impairment was used to exclude individuals not deemed to be cognitively unimpaired on the basis of clinical impression by the examining technician, who was not a licensed neuropsychologist (*N* = 581). This formed our cognitively unimpaired sample (*N* = 1,042). A subset of these individuals were also administered the MMSE as part of their longitudinal involvement in the study, though unlike at LH, there was on average a multi-year difference between DCTclock and MMSE.

For DCTclock development, the baseline DCTclock test of each participant from FHS and LH was combined to form a dataset of presumed cognitively unimpaired and clinically diagnosed cognitively impaired individuals, respectively (*N* = 1,833). The combined dataset was randomly divided into training (*N* = 912) and testing (*N* = 921) sets, stratified by the presence of cognitive impairment to keep the classes balanced in both datasets. Subjects contained within the training set were used solely for development purposes (i.e., building the machine learning models), while subjects within the testing set were used exclusively for statistical and clinical validation. A subset of the testing set was created for participants with both DCTclock and MMSE administered within 18 months of each other (*N* = 591) to enable the benchmark of these two tests. While this time difference is significant and could lead to changes in performance, the most rapid change would be expected from cognitive impaired LH participants who had DCTclock and MMSE within a 2 week period, while the presumed cognitively unimpaired from FHS would likely fluctuate less rapidly making the up to 18 months difference more acceptable. Demographics for both the training and testing data sets are summarized in Table 1.

**Table 1:** Subject demographics for training and testing sets.

**Variable**	**Level**	**Training set**	**Testing set**	***P*** **-value**
Sample size		912	921	
Age [mean (sd)]		69.94 (8.49)	69.88 (8.32)	0.867
Cognitive status [*N* (%)]	Cognitively unimpaired	530 (58.1)	512 (55.6)	0.297
	Cognitively impaired	382 (41.9)	409 (44.4)	
Education [*N* (%)]	College graduate	465 (51.0)	476 (51.7)	0.380
	Some college	221 (24.2)	245 (26.6)	
	HS graduate	192 (21.1)	167 (18.1)	
	Less than HS	34 (3.7)	33 (3.6)	
Gender [*N* (%)]	Female	483 (53.0)	449 (48.8)	0.079
	Male	429 (47.0)	472 (51.2)	
Race [*N* (%)]	Asian	21 (2.3)	21 (2.3)	0.984
	Black/African American	17 (1.9)	16 (1.8)	
	White/Caucasian	861 (95.8)	863 (95.9)	
Ethnicity [*N* (%)]	Hispanic/Latino	13 (1.7)	17 (2.2)	0.568
	Non-Hispanic/Latino	763 (98.3)	754 (97.8)	
MMSE Score (median [IQR])		28 (25, 29)	28 (25, 29)	0.696

### DCT Platform

**Figure 1:**
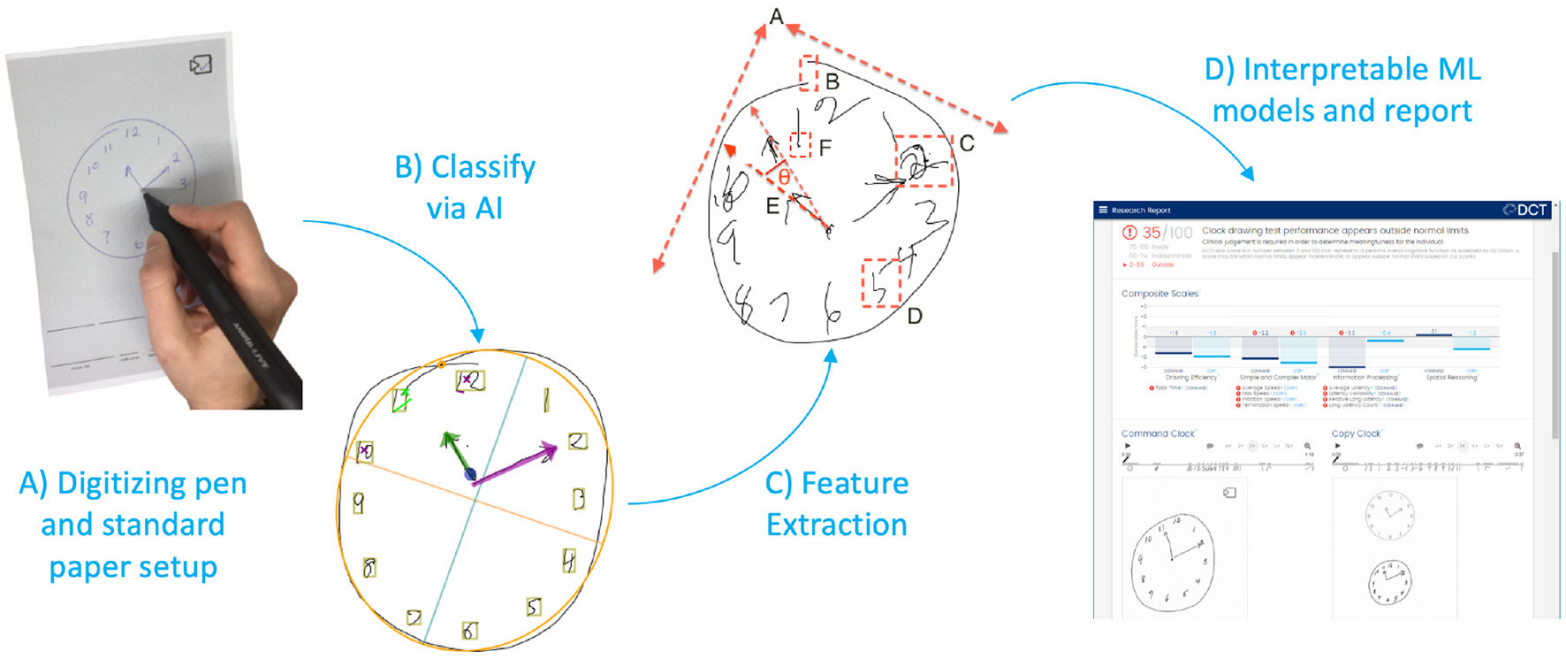
DCTclock automated analysis steps. **(A)** Digitizing pen and standard test paper to capture timestamped-coordinates of drawing motion. **(B)** Time-stamped pen stroke coordinates are classified into drawing symbols. **(C)** Using knowledge from the classified strokes, low-level features are extracted from the drawing process. **(D)** Low-level features are combined into a set of interpretable Composite Scales and an overall score of test performance.

The DCT platform consists of the digitizing ballpoint pen (Figure 1A), a computer application that sends encrypted test data to remote servers where it is analyzed, and a web portal to view and interact with results (Figure 1D). DCTclock is the first cognitive test built on top of the DCT platform. The digital pen functions as an ordinary ballpoint pen while recording its position on the page with considerable spatial (±0.05 cm) and temporal (12 ms) accuracy. The raw data—a set of time-stamped points—is encrypted by the pen and then transferred to a computer that sends the data to DCT servers. The data is securely stored and analysis software is run to analyze the tests. The test administrators and clinicians may log into a web portal to view the tests and results.

### DCTclock Algorithm

#### Pen Stroke Classification for Drawing Understanding

The first step in the analysis consisted of developing an understanding of the drawing by mapping each pen stroke into expected symbols for the representation of a clock—clock face, digits, hands—or as unexpected or unnecessary symbols such as small noise strokes, cross-outs, and overwriting (Figure 1B). We trained a convolutional neural network architecture (23) to recognize these individual symbols by using our own database of labeled symbols from our training set and supplementing it with publicly available digits from MNIST (24). We then developed a multi-pass, multi-layer rules algorithm for pen stroke classification into drawing symbols, that iteratively combined pen strokes based on their spatial and temporal distribution within the drawing process and assigned symbol probabilities to each combination using the trained neural net.

#### Drawing Measurements

Using the pen stroke classification output, measurements are calculated from both the final drawing and the drawing process (Figure 1C). Some measurements derived from the final drawing, such as the correct placement of clock components, are equivalent to measurements used in scoring systems from the CDT; others are novel and include the overall position of the drawing on the page, the total number of strokes, and the lengths of various strokes. Most of the measurements, however, are focused on the drawing process and include total time, different types of latencies, pen speeds and oscillations during various portions of the drawing process, and measures of efficiency in the drawing production.

#### Creating Composite Scales and DCTclock Score for Cognitive Impairment Classification

Measurements were harmonized to produce a collection of meaningful metrics of cognition based on brain-behavior principles. In this process, opaque variables were eliminated through clinician feedback. While this resulted in a small loss of classification accuracy, it produced increased transparency (10). These features, denoted as cognitive features and detailed in Table 2, were designed to have stronger face validity by looking at the drawing or viewing a replay of the drawing process. These measurements were assigned to one of four functional groups Drawing Efficiency, Simple and Complex Motor, Information Processing, and Spatial reasoning.

**Table 2:** Table of DCTclock metrics with definitions and median [IQR].

**DCTclock metric**	**Definition**	**Cognitively unimpaired**		**Cognitively impaired**	
DCTclock score	A number between 0 and 100 that represents a person's overall cognitive function as assessed by DCTclock	86.23 [71.79, 93.68]		39.16 [14.27, 60.00]	
		**Command clock**		**Copy clock**	
		**Cognitively unimpaired**	**Cognitively impaired**	**Cognitively unimpaired**	**Cognitively impaired**
Drawing efficiency	The efficiency the individual demonstrated during the process of drawing each clock. This considers metrics such as time spent relative to properties of the drawing including number of pen strokes, stroke length, and size of the drawing.	0.16 [−0.51, 0.66]	−1.11 [−2.62, −0.06]	0.10 [−0.41, 0.58]	−0.91 [−2.26, 0.07]
Stroke count conformity	The deviation from the expected number of pen strokes in the drawing.	0.00 [0.00, 2.00]	1.00 [0.00, 5.50]	0.00 [0.00, 1.00]	0.00 [0.00, 2.00]
Total time	The total time spent completing the drawing, measured from the first touch of the pen on the paper to the last pen lift off the paper.	34.37 [27.48, 42.98]	44.42 [32.04, 65.01]	26.22 [22.28, 32.49]	32.12 [25.41, 43.58]
Ink length	The sum, in millimeters, of the lengths of all pen strokes used in the drawing.	580.32 [470.2, 694.1]	480.08 [365.47, 637.09]	440.93 [390.92, 495.32]	392.1 [341.4, 464.6]
Drawing Size	The size, in millimeters, of the clock face circle.	80.29 [65.69, 94.16]	64.06 [46.66, 82.06]	59.15 [52.92, 67.71]	50.95 [44.18, 59.52]
Drawing Process Efficiency	A relative measure that combines Ink Length and Total Time.	16.48 [13.06, 20.75]	10.70 [6.93, 15.12]	16.63 [13.11, 20.76]	12.08 [8.42, 16.01]
Noise	A measure of the drawing that includes non-standard pen strokes, cross outs, and overwriting.	0.40 [0.40, 0.42]	0.41 [0.40, 0.44]	0.39 [0.39, 0.42]	0.41 [0.39, 0.44]
**Information processing**	The non-motor cognitive functions used during the drawing process. This considers metrics such as absolute and relative duration of latencies, number of pauses, and relative time spent thinking vs. actively drawing with pen on the paper.	0.19 [−0.49, 0.75]	−0.65 [−1.67, 0.22]	0.16 [−0.59, 0.74]	−0.74 [−1.70, 0.19]
Percent think time	The percentage of the test time spent “thinking” (i.e., holding the pen off the page but not actively drawing) measured from the first touch of the pen on the paper to the last pen lift off the paper.	59.48 [54.01, 65.19]	62.10 [54.78, 69.95]	53.70 [49.00, 58.12]	55.84 [49.20, 62.46]
Average latency	The average duration of the latencies between each pen stroke.	0.84 [0.68, 1.10]	1.18 [0.82, 1.77]	0.62 [0.51, 0.77]	0.80 [0.60, 1.08]
Latency variability	The variability in the latencies throughout the drawing process.	0.99 [0.68, 1.51]	1.62 [0.96, 2.62]	0.49 [0.37, 0.68]	0.79 [0.52, 1.21]
Relative long latency	A measure of the difference between the average latency and the longer latencies within this drawing.	7.26 [5.01, 10.43]	11.62 [6.65, 19.96]	4.20 [3.19, 5.73]	5.98 [4.09, 9.32]
Long latency count	The total number of latencies in the drawing that are notably longer than the normative sample standard.	1.00 [0.00, 1.00]	2.00 [1.00, 3.00]	1.00 [0.00, 2.00]	2.00 [1.00, 4.00]
Longest latency	The duration of the longest latency in the drawing.	4.46 [2.94, 6.84]	6.87 [4.16, 11.64]	1.98 [1.50, 2.83]	3.12 [2.09, 5.37]
**Simple and complex motor**	The graphomotor components involved in the process of drawing each clock. This considers metrics such as pen stroke speeds and oscillatory motion and can be helpful in parsing out motor and non-motor cognitive functions.	0.24 [−0.48, 0.82]	−1.12 [−2.20, −0.04]	0.14 [−0.44, 0.68]	−0.75 [−1.68,0.13]
Percent Ink Time	The percentage of the test time spent actively drawing with the pen on the paper.	40.52 [34.81, 45.99]	37.90 [30.05, 45.22]	46.30 [41.88, 51.00]	44.16 [37.54, 50.80]
Average speed	The average speed of the pen for all pen strokes used during the drawing of the clock face.	127.09 [91.95, 170.8]	81.47 [54.75, 129.92]	100.37 [73.59, 138.40]	78.74 [51.16, 113.3]
Max speed	The maximum speed of the pen on the page during the drawing of the clock face.	208.9 [157.9, 265.2]	158.17 [104.80, 217.74]	168.30 [129.06, 229.47]	140.3 [97.12, 193.2]
Initiation speed	The speed of the pen when beginning to draw the clock face.	109.59 [76.09, 148.5]	76.18 [50.09, 111.70]	85.16 [60.15, 119.44]	68.08 [43.16, 95.27]
Termination speed	The speed of the pen when finishing the clock face.	117.93 [83.36, 166.7]	72.43 [45.29, 116.98]	94.97 [67.99, 138.3]	71.90 [43.42, 110.1]
Oscillatory motion	A measure of how much the motion of the pen deviates from a smooth motion	2.16 [1.67, 2.74]	3.01 [2.35, 3.94]	2.38 [1.93, 2.83]	3.04 [2.47, 3.88]
**Spatial reasoning**	*T*he spatial abilities demonstrated during the drawing process. This considers metrics pertaining to the geometric properties of the drawing including the circularity of the clock circle, placement of clock components, and drawing placement on the page.	0.31 [−0.39, 0.68]	−1.65 [−3.58, −0.21]	0.27 [−0.63, 0.80]	−1.58 [−2.81, −0.49]
Clock face circularity	A measure of the roundness of the clock face circle.	2.38 [2.21, 2.60]	2.59 [2.36, 2.83]	2.36 [2.19, 2.61]	2.55 [2.33, 2.80]
Component placement	A measure of the spatial relationships among the drawing components.	0.29 [0.24, 0.37]	0.52 [0.35, 0.81]	0.32 [0.27, 0.40]	0.49 [0.37, 0.69]
Vertical spatial placement	A measure of the vertical position of the drawing on the page.	6.75 [3.16, 12.12]	10.25 [4.09, 18.12]	10.34 [4.58, 17.52]	19.77 [10.90, 27.98]

For each functional group and for each of the Command and Copy conditions of the clock separately, a composite score was learnt using Lasso regularized logistic regression to classify the Cognitively Impaired and Cognitively Unimpaired groups in our training set. These eight scores were then combined using L1 regularized logistic regression to form a top level DCTclock score.

This analysis results in an easily interpretable hierarchical structure: cognitive problems manifest in performance on cognitive features that individual are easily visually understood and verified, which translates to lower performance on functional domain composites associated with those features, and, in turn, results in a lower DCTclock score.

#### DCTclock Cut Scores

DCTclock score cutoffs that maximize classification accuracy between cognitively unimpaired and cognitively impaired participants were determined by a Receiver Operating Characteristic (ROC) curve analysis on the development sample. Specifically, the cutoff of 60 for impaired classification was determined by the Youden Index, an optimal cutoff point statistic calculated as sensitivity + specificity–1 (25). The cutoff of 75 for Indeterminate classification was determined via a comparative analysis of possible DCTclock cutoffs to established cutoffs used for MMSE classification to facilitate utility in a clinical setting, and was selected to match a cutoff of 28 on the 0–30 MMSE range.

### Statistical Analysis and Validation Procedures

All statistical analyses and validation procedures were conducted on the testing set, using R version 3.4.2.

Demographic variables were summarized by means and standard deviations for normally distributed continuous variables, by median and interquartile range (IQR) for non-normally distributed variables, and by frequencies and percentages for discrete variables. Differences in demographics between the training and testing sets were tested using *t*-tests for normally distributed continuous variables, Wilcoxon Rank Sum tests for non-normally distributed variables, and chi-square tests for discrete variables.

To compare DCTclock to existing clock scoring systems, the following scoring systems were operationalized and applied to our clock test data: MoCA (2), Mini-Cog (26), Manos (27), Royall (28), Shulman (29), Libon (30), Rouleau (31), and Mendez (32). Full details on the operationalization of these scoring systems have been previously published (10).

ROC curves were built for scores of DCTclock and the CDT operationalized scoring systems to measure their ability to distinguish cognitively unimpaired and cognitively impaired participants in the testing set (*N* = 905). ROC curves were also built for participants in the testing set with both DCTclock scores and MMSE scores within 18 months of each other (*N* = 591). The area under the ROC curve was calculated for all curves and differences in AUC between DCTclock and operationalized scoring systems and relation between DCTclock and MMSE scores were tested using the paired non-parametric test presented by DeLong et al. (33). Sensitivities and specificities between DCTclock scores and MMSE scores were compared at the optimal cutoff point, determined by the Youden Index (27).

For each metric developed, differences between cognitively unimpaired and cognitively impaired groups were tested using the Wilcoxon Rank Sum test, adjusting for multiple comparisons using the Bonferroni-Holm method (34). Additionally, a subgroup analysis was conducted using primary consensus diagnosis for subjects diagnosed with aMCI and AD and compared to cognitively unimpaired. Overall group differences for both DCTclock and MMSE were tested using the Kruskal-Wallis test; *post-hoc* analyses were conducted using the Wilcoxon Rank Sum test, adjusting for multiple comparisons using the Bonferroni-Holm method.

Construct validity of DCTclock was determined by correlational analyses. For a given NP test, the Pearson correlation coefficient was calculated for individuals in the testing set who took a DCTclock test and the specific NP test within 10 days of each other. Thus, the sample size (Table 3) for each NP test varied, as not all participants were administered all NP tests within that window.

**Table 3:** Correlation of DCTclock scores to standard neuropsychological test scores.

**Neuropsychological test** **(***n***)**	**Correlation,** ***r***^**2**^ **(95% CI)**
RBANS (256)	0.59 (0.51, 0.67)
MoCA (85)	0.55 (0.39, 0.69)
MMSE (772)	0.60 (0.55, 0.64)
Trails A (400)	0.66 (0.60, 0.71)
Trails B (357)	0.60 (0.53, 0.66)
Hooper (340)	−0.51 (−0.58, −0.43)
Beck depression inventory (386)	−0.14 (−0.23, −0.04)

## Results

### Demographics

Demographics for both the training and testing data sets are summarized in Table 3. There were no significant differences in demographic variables between the training and testing groups. The mean age for both sets was 69.9, the majority were non-Hispanic, white, college graduates. For both sets, the median MMSE score was 28. Approximately 43% were cognitively impaired in each dataset.

### DCTclock Metrics

The list of developed metrics and their definitions can be found in Table 2, along with the median and IQR of each metric across both the cognitively unimpaired and cognitively impaired groups. Every metric was shown to be significantly different across the two groups at *p* < 0.01.

### Validation of DCTclock

**Figure 2:**
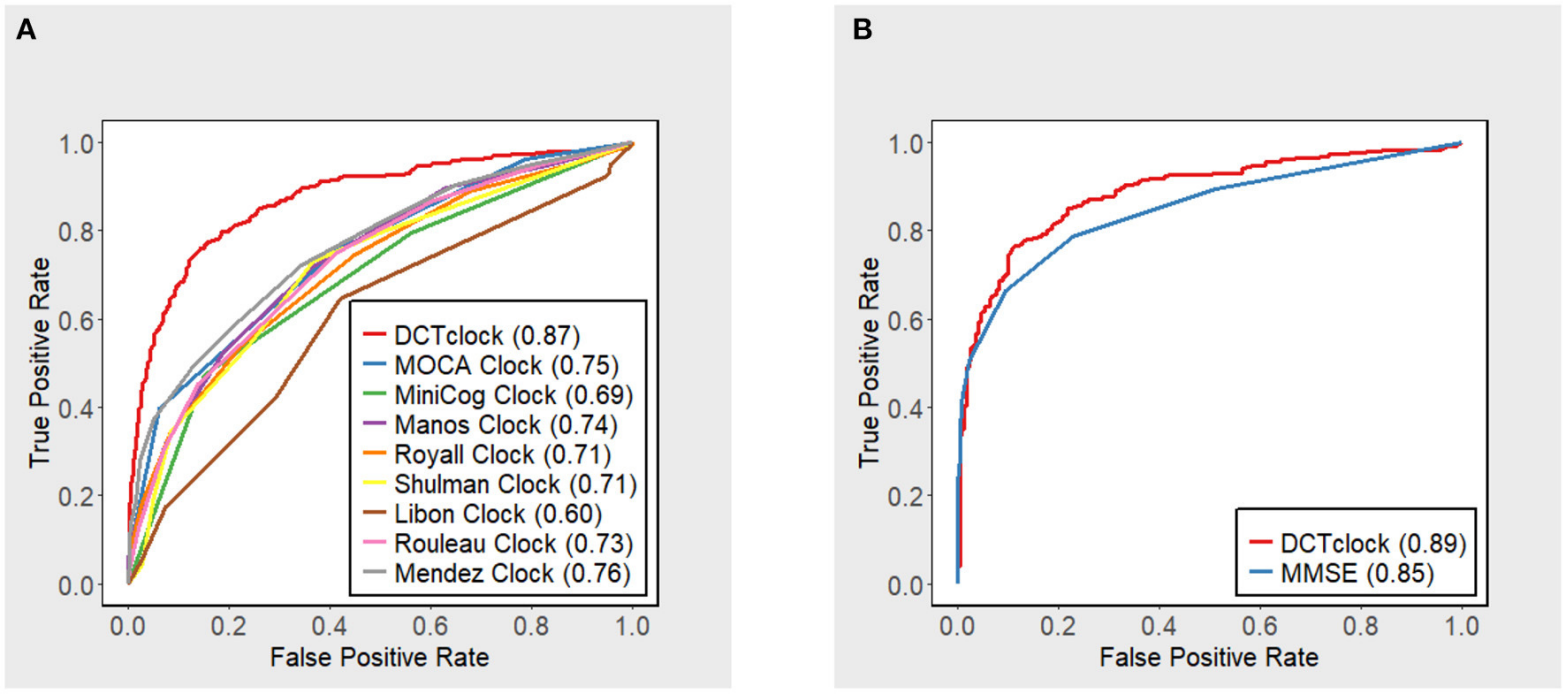
ROC curves with accompanying AUC estimates for DCTclock scores and **(A)** operationalized clock scoring systems and **(B)** MMSE.

DCTclock overall score accuracy measures were compared with the operationalizations of other clock scoring systems using the overall testing set (*N* = 921) (Figure 2A). Results indicate that DCTclock score has a significantly higher AUC compared to the operationalized scoring systems. To compare the discriminatory ability of DCTclock to that of MMSE, ROC analysis was conducted on the subset of the testing set who were administered both DCTclock and the MMSE (*N* = 591). Construction of a ROC curve for DCTclock scores indicate an AUC of 0.89, while the MMSE showed a statistically significantly lower AUC of 0.85 (*p* = 0.029) (Figure 2B). The sensitivity and specificity of DCTclock at the optimal cutoff of 60 (Youden Index) was 0.74 and 0.90, respectively, while the MMSE had a lower sensitivity (0.67) and comparable specificity (0.90) at the optimal cutoff of 27.5 (i.e., <28 vs. ≥28).

**Figure 3:**
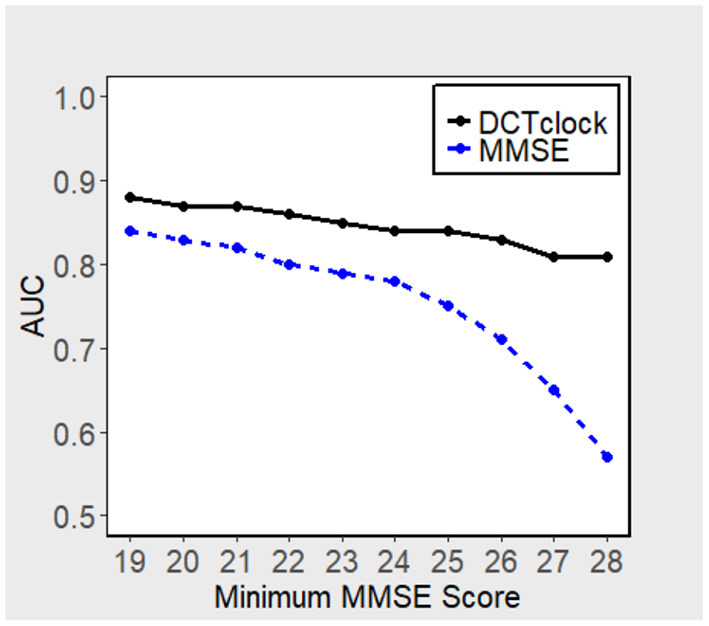
AUCs of DCTclock and MMSE, subset using minimum MMSE score.

Figure 3 displays the performance, in terms of AUC, of DCTclock for different population subsets based on minimum MMSE score (i.e., subset of subjects who scored > 19, 20, 21, etc. on the MMSE). DCTclock's AUC is >0.80, independently of the subset being analyzed.

Using the primary diagnoses provided by LH, probable AD, aMCI, and the FHS cognitively unimpaired subgroups were summarized by MMSE score and DCTclock score. MMSE scores differed significantly (*p* < 0.001) between the groups, with median [Q1, Q3] of 29 [29, 30], 27 [25.25, 29], and 21 [19, 23] for the cognitively unimpaired, aMCI, and probable AD groups, respectively, and some did show the previously reported ceiling effect [40, 41]. DCTclock score also differed significantly (*p* < 0.001) between cognitively unimpaired (86.23 [71.79, 93.68]), aMCI (48.50 [38.80, 68.40]), and probable AD (15.45 [6.36, 39.94]). Wilcoxon Rank Sum tests showed significant differences between cognitively unimpaired and aMCI, as well as between aMCI and AD, individually, after adjusting for multiple comparisons.

Within the LH subjects, DCTclock Score correlates well with the RBANS, MoCA, MMSE, Trails A, Trails B, and Hooper tests. This indicates that, in this sample, there is significant agreement between the DCTclock algorithm and these standard Neuropsychological tests. Conversely, DCTclock Score does not show a significant correlation with the Beck Depression Inventory and the Geriatric Depression Scale; the low (close to zero) correlations indicate that DCTclock is not measuring indices associated with depression or emotional state, but rather are specific to cognition.

## Discussion

We report on the successful development of sensitive, impactful cognitive tests leveraging the combination of well-known clinical science with state-of-the-art AI techniques. We demonstrate that it is possible to create an easy to use and patient-friendly test that provides sensitive and clinician-usable information by combining precise behavior capture on large well-defined population samples, a first layer of AI algorithms for understanding behavior (i.e., mapping pen strokes to drawing symbols), and a second layer that uses more interpretable machine learning models in conjunction with clinician insights to create transparent and easily-understood metrics. This approach, illustrated here with the DCTclock, can be applied to other cognitive tasks and other cognitive domains, and promises to be particularly impactful when multi-modal signals are integrated to provide a more sensitive marker of brain function.

Our approach enables the precise analysis of cognitive behavioral characteristics that are difficult for clinicians to see and impossible to measure accurately without advanced technologies. The CDT has been in clinical use for more than 50 years and has diverse scoring systems. Yet, comparison of DCTclock metrics (Table 2) to eight of the most commonly used traditional scoring system reveals negligible overlap in the elements that are scored. Our approach offers insights into cognition that are simply not possible using non-digital data and either clinician or AI approaches alone. A first reason is that the process of solving the task previously received no formal evaluation, and the time spent not actively drawing was simply not recorded; in our sample, this accounts for between 53 and 62% of the testing time (Table 2, Percent Think Time). Second, a data-driven approach using AI enables the learning of novel measures of cognition from the data, and weighting them so as to optimize detection of cognitive impairment. This is enabled by large sample sizes of well-defined and precisely characterized subjects for both training and testing sets; a key strength of the development of DCTclock was the opportunity to obtain subject data from research projects, such as the Framingham Heart Study that was integral to this work, underscoring the importance of data sharing and harmonization of large data repositories. Finally, instead of using black-box AI models which would simply produce an overall score from the raw test data, splitting the analysis into two steps with a focus on clinical interpretability of the results through hierarchical construction of scores from clinically meaningful variables can enable deeper clinical insights and, ideally, assessment of the source of impairment.

Validation data shows accuracy, as measured by AUC, sensitivity, and specificity, substantially greater than the standard CDT and MMSE, as well as the ability to distinguish between cognitively unimpaired, aMCI, and probable AD. Strong correlations with standard neuropsychological screening tests, such as the RBANS and MoCA, underscore the ability of this methodology to reliably capture cognitive information in less time.

From a patient perspective, this methodology allows for a rapid test that is unintimidating and well-tolerated. The median time for test completion is ~35 and 25 s for command and copy clocks, respectively, with those times increasing to 44 and 32 s in our cognitively impaired group (Table 2, Total time). Combined with the very short test instructions, this enables the total test time to expect to be below 2 min. Beyond the workflow efficiency, this could enable increased sensitivity in detection of subtle cognitive change that could otherwise be missed due to increased stress and fatigue that may be inherent in more intensive and time-consuming tests. For test administrators, the testing protocol remains largely unchanged, with use of standard test administration procedures but complemented with a fully automated and objective scoring system, obviating subjective assessment.

Importantly, the results demonstrate potential for DCTclock in differential diagnosis. The lack of correlation with the Beck Inventory demonstrates that the DCTclock algorithm is detecting cognitive issues exclusive of depression. While more validation will be needed, this could potentially lead to improved specialist referrals, especially from the perspective of primary care providers hesitant between a referral to neurology or psychiatry.

There are some limitations and potential improvements to our analysis. First, several race and ethnic groups were under-represented in our samples. DCTclock is designed to be applicable to diverse populations and refining DCTclock algorithm performance in widely diverse populations is important for future studies. Second, although comments from early adopters have been positive, additional studies are also needed to assess the feasibility of using DCTclock in diverse clinical settings and to evaluate impact on efficiency, efficacy, and patient outcomes. Third, the dataset was collected over a decade, limiting our definition of AD, and aMCI groups to an older non-biomarker standard. AD along with other neurocognitive conditions are moving toward a biomarker-based definition of disease detection and progression (35). Current literature suggest that a substantial proportion of individuals 55 years or older who are not cognitively impaired as assessed from standard cognitive tests still have evidence of amyloid and tau deposition on PET or abnormal CSF consistent with neurodegenerative disease, including AD. Therefore, a portion of our cognitively unimpaired population may be AD biomarker positive, contaminating our sample and possibly reducing sensitivity. Conversely, those in the probable AD/aMCI groups may not have biomarker positive AD. It is thus important for future validation of DCTclock to study the detection of biomarker positive against biomarker negative samples and to focus on the ability to detect subtle cognitive impairment associated with known diagnostic biomarker measures. Future plans include biomarkers studies focusing on measuring the association of DCTclock metrics with PET amyloid and tau. In fact, one such validation study comparing DCTclock to PET global amyloid, PET global tau, and the A4 Preclinical Alzheimer Cognitive Composite (PACC) has already obtained interesting results (15), showing that DCTclock performance was strongly associated with both amyloid and tau deposition in both unimpaired and subtly impaired individuals as measured by the PACC, and also correlated well with the PACC. Thus, we believe that this novel integrative model of clinician, artificial intelligence, and technology provide exciting opportunities for scientific advancement in the understanding cognitive change that may occur in early stage neurodegenerative disorders.

## Data Availability Statement

The datasets presented in this article are not readily available because data access permission must be requested with the Framingham Heart Study and Lahey Hospital and Medical Center. Requests to access the datasets should be directed to https://biolincc.nhlbi.nih.gov/studies/framoffspring/.

## Ethics Statement

The studies involving human participants were reviewed and approved by WIRB (IRB Tracking Number 20160721). The patients/participants provided their written informed consent to participate in this study.

## Author Contributions

WS-M model development, contributed to statistical analyses, and led the writing of the paper. RD contributed to model development, provided input in statistical analyses, and helped with writing and editing. DP contributed to model development, Lahey Hospital and Medical Center data supervision and interpretation, provided input in statistical analyses and clinical interpretation, and helped with writing and editing. BS contributed to statistical analyses and helped with writing and editing. RA Framingham Heart Study data supervision and interpretation, provided input in statistical analyses and clinical interpretation, and helped with editing. AP-L contributed to clinical interpretation of the results and helped with editing. All authors contributed to the article and approved the submitted version.

## Funding

Support for this work came in part from: The Robert E. Wise Research and Education Institution, Defense Advanced Research Projects Agency Contract D13AP00008, National Science Foundation Award IIS-1404494, National Institute of Neurological Disorders and Stroke Grants R01-NS17950, K23-NS60660, and R01-NS082386, National Heart, Lung, and Blood Institute Contract N01-HC25195, National Institutes on Aging Grants R01 AG0333040, AG16492, AG08122, and AG054156, National Institute of Neurological Disorders and Stroke NS017950; National Institute on Mental Health Grant RO1-MH073989, IH/NCATS Clinical and Translational Science Award to the University of Florida UL1TR000064, NHLBI, Framingham Heart Study, (NHLBI/NIH contract #HHSN268201500001I), the Boston University School of Medicine, the University of Florida Movement Disorders and Neurorestoration, National Parkinson Foundation, and Pfizer.

## Conflict of Interest

WS-M was founding VP of AI and Analytics at Digital Cognition Technologies Inc. (acquired by Linus Health Inc.), VP of AI and Analytics at Linus Health Inc., and is now an advisor at Linus Health Inc. DP and RD were founders of Digital Cognition Technologies Inc. BS was a Principal Statistician at Digital Cognition Technologies Inc. AP-L was a co-founder of Linus Health and TI Solutions AG; serves on the scientific advisory boards for Starlab Neuroscience, Magstim Inc., and MedRhythms; and is listed as an inventor on several issued and pending patents on the real-time integration of non-invasive brain stimulation with electroencephalography and magnetic resonance imaging. AP-L was partly supported by the National Institutes of Health (R24AG06142, and P01 AG031720), the National Science Foundation, and the Barcelona Brain Health Initiative primarily funded by La Caixa. RA was scientific advisor to Signant Health and a scientific consultant to Biogen.

## Publisher's Note

All claims expressed in this article are solely those of the authors and do not necessarily represent those of their affiliated organizations, or those of the publisher, the editors and the reviewers. Any product that may be evaluated in this article, or claim that may be made by its manufacturer, is not guaranteed or endorsed by the publisher.
